# Dietary bile acids supplementation decreases hepatic fat deposition with the involvement of altered gut microbiota and liver bile acids profile in broiler chickens

**DOI:** 10.1186/s40104-024-01071-y

**Published:** 2024-08-13

**Authors:** Minghui Wang, Kelin Li, Hongchao Jiao, Jingpeng Zhao, Haifang Li, Yunlei Zhou, Aizhi Cao, Jianmin Wang, Xiaojuan Wang, Hai Lin

**Affiliations:** 1 College of Animal Science and Technology, Shandong Provincial Key Laboratory of Animal Biotechnology and Disease Control and Prevention, Key Laboratory of Efficient Utilization of Non-Grain Feed Resources (Co-Construction By Ministry and Province), Ministry of Agriculture and Rural Affairs, Shandong Agricultural University, No. 61, Daizong Street, Taian, 271018 Shandong P. R. China; 2 College of Life Sciences, Shandong Agricultural University, No. 61, Daizong Street, Taian, 271018 Shandong P. R. China; 3 College of Chemistry and Material Science, Shandong Agricultural University, No. 61, Daizong Street, Taian, 271018 Shandong P. R. China; 4 Shandong Longchang Animal Health Products Co., Ltd., Jinan, P. R. China

**Keywords:** Bile acids, Broiler chickens, Gut microbiota, Hepatic fat deposition, Liver bile acid profile

## Abstract

**Background:**

High-fat diets (HFD) are known to enhance feed conversion ratio in broiler chickens, yet they can also result in hepatic fat accumulation. Bile acids (BAs) and gut microbiota also play key roles in the formation of fatty liver. In this study, our objective was to elucidate the mechanisms through which BA supplementation reduces hepatic fat deposition in broiler chickens, with a focus on the involvement of gut microbiota and liver BA composition.

**Results:**

Newly hatched broiler chickens were allocated to either a low-fat diet (LFD) or HFD, supplemented with or without BAs, and subsequently assessed their impacts on gut microbiota, hepatic lipid metabolism, and hepatic BA composition. Our findings showed that BA supplementation significantly reduced plasma and liver tissue triglyceride (TG) levels in 42-day-old broiler chickens (*P* < 0.05), concurrently with a significant decrease in the expression levels of fatty acid synthase (FAS) in liver tissue (*P* < 0.05). These results suggest that BA supplementation effectively diminishes hepatic fat deposition. Under the LFD, BAs supplementation increased the BA content and ratio of Non 12-OH BAs/12-OH BAs in the liver and increased the *Akkermansia* abundance in cecum. Under the HFD, BA supplementation decreased the BAs and increased the relative abundances of chenodeoxycholic acid (CDCA) and cholic acid (CA) in hepatic tissue, while the relative abundances of *Bacteroides* were dramatically reduced and the *Bifidobacterium*, *Escherichia*, and *Lactobacillus* were increased in cecum. Correlation analyses showed a significant positive correlation between the *Akkermansia* abundance and Non 12-OH BA content under the LFD, and presented a significant negative correlation between the *Bacteroides* abundance and CA or CDCA content under the HFD.

**Conclusions:**

The results indicate that supplementation of BAs in both LFD and HFD may ameliorate hepatic fat deposition in broiler chickens with the involvement of differentiated microbiota–bile acid profile pathways.

**Graphical Abstract:**

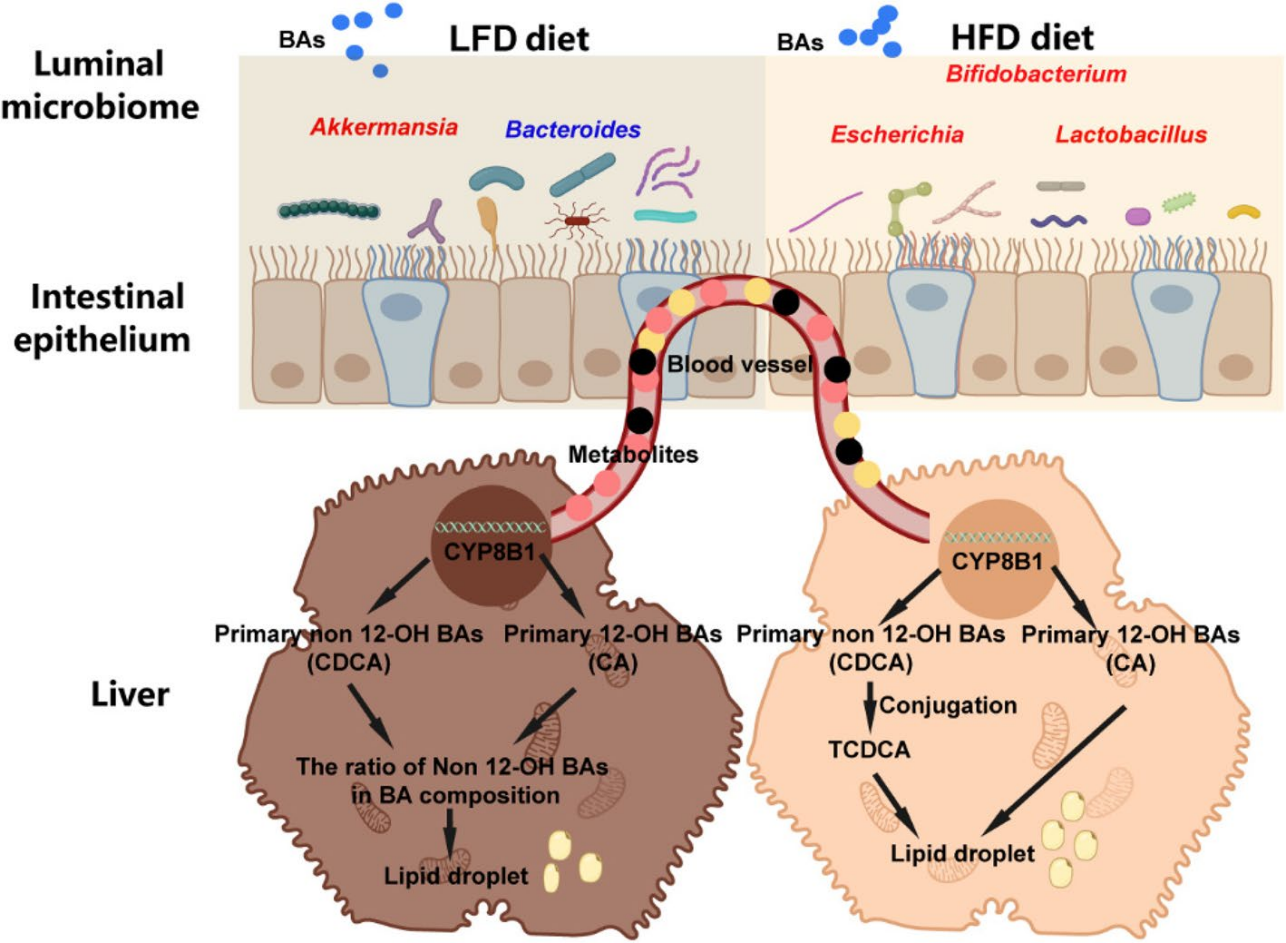

## Background

Broiler chickens are favored for their rapid growth, high feed conversion rates, low resource consumption, and short production cycles, offering economic and environmental advantages [1]. Fast-growing broiler chickens with their exceptional growth potential are highly sought after in the poultry market. This is achieved by optimizing feed conversion ratio (FCR) through the addition of high-fat diet (HFD), sometimes reaching fat levels as high as 6%–10% [2–4]. However, when physically restricted chickens (primarily occurring in caged broiler chickens) are allowed to consume HFD, disruptions occur in fat metabolism, particularly in liver tissue, leading to fat accumulation in liver cells and increased sudden death [5]. Fatty liver syndrome is a mainly non-communicable metabolic disease occurring in the poultry industry, as 90% of fatty acids in poultry are synthesized in the liver [6]. Fatty acid synthesis involves the conversion of non-lipid precursor molecules, such as glucose and some amino acid metabolites, into fatty acids, ultimately forming triglyceride (TG) [7]. The process primarily involves acetyl-CoA, catalyzed by synthetic enzymes including acetyl-CoA carboxylase (ACC) and fatty acid synthase (FAS), through a series of intermediate reactions regulated by sterol regulatory element-binding proteins (SREBPs), leading to the synthesis of fatty acids in the cytosol [8]. The process of fat mobilization involves the gradual breakdown of TG into non-esterified fatty acids (NEFAs) by lipases [9]. Maintaining a dynamic balance of lipid is achieved through the stable regulation of both fat synthesis and fat breakdown processes within the body [10].

Bile acids (BAs) are one of the main components of bile synthesized in the liver from cholesterol through the "classic pathway" mediated by cholesterol 7α-hydroxylase (CYP7A1) and the "alternative pathway" mediated by sterol 27-hydroxylase (CYP27A1) playing a crucial role in organismal lipid metabolism [11]. The specific structure makes BAs potent emulsifiers, reducing the interfacial tension between oil and water phases, emulsifying lipids into small micelles, increasing the surface area for lipid digestion [12]. Studies have shown that long-term consumptions of HFD significantly decrease liver and serum BA levels in mice, primarily due to the inhibition of hepatic BA synthesis and reduced efficiency of BAs reabsorption in the enterohepatic circulation [13]. This alteration was also companied by the changed compositions and structures of intestinal microbiota in mice, leading to lipid metabolism disorders [14]. However, supplementing 60 mg/kg BAs to the diet of laying hens reduced the hepatic tissue TG content, indicating that BAs can regulate lipid metabolism in poultry liver tissue [15]. The classic pathway responsible for at least 75% primary BAs produces cholic acid (CA) and chenodeoxycholic acid (CDCA) after the actions of cytochrome P450 family 8 subfamily B member 1 (CYP8B1) and CYP27A1 [16, 17]. The alternative pathway synthesizes CDCA through CYP27A1, followed by the 7α-hydroxylation of 27-hydroxycholesterol and other oxysterols by CYP7B1. Furtherly, CYP8B1 determines the abundance of CA and CDCA in alternative pathway produced Non-12-OH BAs, in which CDCA was the major component [16]. These BAs exhibit species differences, such as mice primarily producing muricholic acid (MCA), bears producing ursodeoxycholic acid (UDCA), and pigs producing hyodeoxycholic acid (HCA) [18]. In the intestine, enzymes from intestinal microbiota further metabolize BAs to secondary BAs. For example, primary conjugated BAs can be deconjugated by bile salt hydrolase (BSH) to free BAs, which are then 7α-dehydroxylated to form corresponding secondary BAs (DCA and LCA) [19]. Therefore, the interactions between microbiota and BAs may play key roles in alleviating fatty liver induced by the BAs or gut microbiota interventions. At the distal ileum, most unconjugated BAs including a small fraction of free BAs and transformed secondary BAs are reabsorbed into enterocytes and enter the portal circulation through basolateral BA transporters, returning to the liver via the bloodstream [20].

Hence, it is imperative to investigate whether the fatty liver stemming from HFD in broiler chickens can be mitigated through the inclusion of exogenous BAs. Meanwhile, exploring whether the impact of exogenous BAs remains consistent across broiler chickens fed diets with different fat levels and the potential mechanism from the perspective of microbiota are crucial. In this study, both LFD and HFD were employed to evaluate the repercussions of incorporating BAs on the lipid metabolism, hepatic BA composition, and gut microbiota in broiler chickens.

## Methods

### Animal management and dietary treatment

The animal experiment conducted in this study was reviewed and approved by the Institutional Animal Care and Use Committee of Shandong Agricultural University (No. 2001002) and performed in accordance with the "Guidelines for Experimental Animals" outlined by the Ministry of Science and Technology, Beijing, P. R. China. The BAs (purity 97.1%) used in this research were supplied by Shandong Longchang Animal Health Product Co., Ltd. (Dezhou, China) in which the BAs were extracted from pig bile through a process including saponification, decolorization, acidification, purification, and drying [21]. The quantity of each bile acid was determined using high-performance liquid chromatography (Q/371425SLC020-2023, standard for bile acid by Shandong Longchang Animal Health Product Co., Ltd.). The combined contents of hyocholic acid and hyodeoxycholic acid accounted for 77.2%, while chenodeoxycholic acid accounted for 19.9% (LC(2022)0622, Shandong Longchang Animal Health Product Co., Ltd.).

A total of 640 one-day-old broiler chicks (Arbor Acres) were purchased from a local hatchery (Dabao Breeding Ltd., Taian, China) and randomly allocated to 32 pens with 20 chicks each. The chicks were further divided into 4 groups and each group had 8 pens. The 4 groups were subjected to one of the following treatments: low-fat diet (2.22% EE from d 1 to 21 and 4.79% EE from d 22 to 42, LFD), LFD supplemented with 60 mg/kg BAs (LFD + BAs), HFD (5.40% EE from d 1 to 21 and 7.65% EE from d 22 to 42, HFD), or HFD supplemented with 60 mg/kg BAs (HFD + BAs). The diets were formulated according to the recommendations of the National Research Council (NRC, 1994) [22]. The experimental design is presented in Table 1. The ingredients and nutrient composition are provided in Table 2.

**Table 1:** Layout of experimental design

Diet	BAs	
	0 mg/kg	60 mg/kg
Low-fat diet	LFD	LFD + BAs
High-fat diet	HFD	HFD + BAs

**Table 2:** Ingredients and nutrient composition of the experimental diets (as-fed basis)

Ingredients, %	1–21 d		22–42 d	
	LFD	HFD	LFD	HFD
Corn (8.0% CP)	61.58	49.68	66.45	54.56
Soybean meal (43% CP)	22.00	25.48	16.46	21.72
Corn gluten meal (59% CP)	10.04	7.03	10.00	6.00
Wheat	1.00	9.38	1.00	8.42
Soybean oil	0.79	3.99	1.60	4.92
Limestone	1.31	1.30	1.42	1.42
CaHPO_4_	1.68	1.61	1.56	1.48
NaCl	0.29	0.28	0.28	0.26
Lysine (99%)	0.33	0.25	0.38	0.27
Methionine (98%)	0.13	0.15	0.07	0.10
Threonine (98%)	0.09	0.09	0.08	0.07
Choline chloride (50%)	0.26	0.26	0.20	0.20
Vitamin premix^1^	0.30	0.30	0.30	0.30
Mineral premix^2^	0.20	0.20	0.20	0.20
Nutrient composition				
Crude protein, %	21.00	21.00	19.00	19.00
Metabolic energy, kcal/kg	3,000	3,000	3,100	3,100
Calcium, %	0.90	0.90	0.90	0.90
Available phosphorus, %	0.43	0.43	0.40	0.40
Lysine, %	1.10	1.10	1.02	1.02
Methionine, %	0.50	0.50	0.42	0.42
Met + Cys, %	0.85	0.85	0.74	0.74
Threonine, %	0.90	0.90	0.80	0.80
EE, %^3^	2.22	5.40	4.79	7.65
NFE, %	52.82	49.40	54.54	51.03
Crude fiber, %	2.30	3.00	2.10	2.80

^1^Vitamin premix provides the following per kg of diet: VA, 8,000 IU; VD_3_, 3,000 IU; VE, 20 IU; VK, 2 mg; VB_1_, 4 mg; riboflavin, 8 mg; D-pantothenic acid, 11 mg; VB_5_, 40 mg; VB_6_, 4 mg; VB_12_, 0.02 mg; biotin, 0.15 mg; folic acid, 1 mg; choline, 700 mg

^2^Mineral premix provides the following per kg of diet: Fe (as ferrous sulfate), 80 mg; Zn (as zinc sulfate), 75 mg; Mn (as manganese sulfate), 80 mg; Cu (as copper sulfate) 10 mg; I (as potassium iodide), 0.40 mg; Se (as sodium selenite), 0.30 mg

^3^Measured value

### Growth performance

The body weight (BW) of the broiler chickens was recorded on d 1, 21, and 42. Subsequently, the average daily gain (ADG), average daily feed intake (ADFI), and FCR were calculated.

### Sample collection

On d 21 and 42, one bird from each pen was selected for sampling. The blood samples were collected from wing veins and then centrifuged at 3,000 × *g* for 10 min at 4 °C to obtained the plasma stored at –20 °C until analysis. The broiler chickens were euthanized by cervical dislocation. Samples of the liver, small intestine, and cecal content were collected and stored at –80 °C until analysis.

### Plasma biochemical indices

The concentrations of total BAs (TBA), glucose (GLU), total cholesterol (TCHO), triglycerides (TG), high-density lipoprotein cholesterol (HDL-CHO), low-density lipoprotein cholesterol (LDL-CHO), aspartate transaminase (AST), and alanine aminotransferase (ALT) were measured using commercially available kits (Nanjing Jiancheng Bioengineering Institute, Jiangsu, China).

### Sequencing and analysis of 16S rRNA gene

Microbial DNA was extracted from the cecal samples using the E.Z.N.A.^®^ DNA kits (Omega Biotek, Norcross, GA, USA) according to the manufacturer’s protocol. The final concentrations and purities of the DNA samples were determined using a NanoDrop 2000 UV–vis spectrophotometer (Thermo Scientific, Wilmington, USA), and the DNA quality was further assessed by 1% agarose gel electrophoresis. The V3–V4 hypervariable regions of the bacterial 16S rRNA gene were amplified using the GeneAmp 9700 thermocycler PCR system (ABI, USA) with the primers 338F (5'-ACTCCTACGGGAGGCAGCAG-3') and 806R (5'-GGACTACHVGGGTWTCTAAT-3') and FastPfu Polymerase. The PCR products were extracted with the 2% agarose gel, further purified using the AxyPrep DNA Gel Extraction kit (Axygen Biosciences, CA, USA), and quantified using QuantiFluor™-ST (Promega, USA) according to the manufacturer’s instructions. Purified amplicons were pooled at equimolar concentrations, established Illumina libraries, and then subjected to paired-end sequencing (2 × 300) on an Illumina MiSeq platform (Illumina, San Diego, USA) following standard protocols. Data analysis was performed using the "Atacama soil microbiome tutorial" of QIIME2docs along with customized program scripts (https://docs.qiime2.org/2019.1/). Diversity metrics were calculated using the core-diversity plugin within QIIME2, including the observed OTUs, Chao1 richness estimator and Shannon diversity index to estimate the microbial diversity. The beta diversity distance measurements, such as Bray Curtis, unweighted UniFrac, and weighted UniFrac, were used to investigate the structural variation of the microbial communities across the samples, which were then visualized through principal coordinate analysis (PCoA). Spearman correlation analysis was performed to investigate the relationship between microbiota and the BAs.

### Measurement of liver BAs

Liver BAs were quantified using ultra-performance liquid chromatography-tandem mass spectrometry (UPLC-MS/MS). Briefly, tissues (approximately 100 mg) were extracted with 1 mL of methanol using ultrasonic assistance. The resulting methanol extracts were centrifuged, filtered, and quantified using the UPLC-MS/MS system according to the established protocols [23]. Each individual bile acid was identified by comparing its retention time with the corresponding reference standard substance.

### Real-time quantitative PCR

Total RNA was extracted from cells or tissues using total RNA kits (OMEGA, Connecticut, USA). The quantity and quality of the isolated RNA were measured using a spectrophotometer (UV-2450; Shimadzu Corporation, Kyoto, Japan). Reverse transcription was performed using the commercial cDNA synthesis kits (Roche, Basel, Switzerland) with 1 μg of RNA for each sample. To determine the mRNA expressions of the target genes, cDNA was amplified using a FastStart Universal SYBR Green Master Mix (Roche). Primers were designed using Primer 7 software (SPS Inc., CA, USA) and synthesized by Sangon Biotech (Shanghai) Co., Ltd. (Shanghai, China). Real-time PCR was conducted on a Q5 Real-Time PCR System (Applied Biosystems, CA, USA). *GAPDH* was used as the reference gene for normalization. The primer sequences are listed in Table 3. The expression levels were quantified using the comparative CT method (2^−ΔΔCt^).

**Table 3:** Gene-specific primers used for chicken gene expression analysis

Gene	Sequences (5′→3′)	Accession No.	Product size, bp
*GAPDH*	F-CTACACACGGACACTTCAAG R-ACAAACATGGGGGCATCAG	NM_204305.1	244
*ACC*	F-AATGGCAGCTTTGGAGGTGT R-TCTGTTTGGGTGGGAGGTG	XM_015295697.2	136
*ADPN*	F-ACCCAGACACAGATGACCGTT R-GAGCAAGAGCAGAGGTAGGAGT	XM_015276846.2	239
*ADPR1*	F-GGAGAAGGTTGTGTTTGGGATGT R-TGGAGAGGTAGATGAGTCTTGGC	NM_001031027.1	218
*ADPR2*	F-ACACACAGAGACTGGCAACATC R-CCCAAGAAGAACAATCCAACAACC	NM_001007854.1	144
*AMPK*	F-GGGACCTGAAACCAGAGAACG R-ACAGAGGAGGGCATAGAGGATG	NM_001039605.1	215
*ASBT*	F-TCACAGCCTTCTTGCTTTCA R-TGTCACCATCCACCCAGTAG	NM_001319027.1	126
*ATGL*	F-AAGTCCTGCTGGTCCTCTCCTTG R-AGTGTTGTCCTCCATCTGGTCCTC	NM_001113291.1	94
*BSEP*	F-TGGAATAGAGCGTGGCTTTT R-CATTGGCAGTCATCTCAGGA	XM_015289699	121
*C/EBPα*	F-TGGACAAGAACAGCAACGAG R-AGCTCCAGCACCTTCTGCT	NM_001031459.1	118
*CPT1*	F-GGAGAACCCAAGTGAAAGTAATGAA R-GAAACGACATAAAGGCAGAACAGA	XM_015286798.2	135
*CYP7A1*	F-GATCTTCCCAGCCCTTGTGG R-AGCCTCTCCCAGCTTCTCAC	NM_001001753.1	82
*FABP4*	F-TGAAGCAGGTGCAGAAGT R-CAGTCCCACATGAAGACG	NM_204290.1	149
*FAS*	F-CTATCGACACAGCCTGCTCCT R-CAGAATGTTGACCCCTCCTACC	NM_205155.3	107
*FATP1*	F-TCAGGAGATGTGTTGGTGATGGAT R-CGTCTGGTTGAGGATGTGACTC	NM_001039602.2	138
*FXR*	F-AGTAGAAGCCATGTTCCTCCGTT R-GCAGTGCATATTCCTCCTGTGTC	NM_204113.2	182
*LPL*	F-CAGTGCAACTTCAACCATACCA R-AACCAGCCAGTCCACAACAA	XM_015280414.2	150
*ME*	F-TGCCAGCATTACGGTTTAGC R-CCATTCCATAACAGCCAAGGTC	NM_204303.1	175
*NTCP*	F-AGACAGGGATGGTTGTGCTT R-CTGAGGGGAGATGGTGATGT	XM_015287931.1	106
*PPARα*	F-AGACACCCTTTCACCAGCATCC R-AACCCTTACAACCTTCACAAGCA	XM_025150258.1	167
*PPARγ*	F-CCAGCGACATCGACCAGTT R-GGTGATTTGTCTGTCGTCTTTCC	XM_015292933.2	145
*SREBP1*	F-GCCCTCTGTGCCTTTGTCTTC R-ACTCAGCCATGATGCTTCTTCC	NM_204126.2	130

### Statistical analysis

Prior to analysis, all data were tested for homogeneity and a normal distribution of variances among the treatments using the UNIVARIATE procedure. For variables ADFI, BWG, FCR, organ index, plasma parameters, mRNA expression levels and liver BA composition, a two-way ANOVA model (version 8e, SAS Institute, Cary, NC, USA) was used to assess the main effects of diet and BAs, as well as their interaction. A significance level of *P* < 0.05 is considered the indication of statistical significance. 0.05 < *P* < 0.1 means a tendency to difference.

## Results

### Effects of BAs on the growth performance in broiler chickens fed diets with different fat levels

Table 4 depicts the impact of BA supplementation and dietary fat levels on the growth performance of broiler chickens. Growth performance of broiler chickens from d 1 to 21 showed that the HFD led to a significant increase in ADFI during this period (*P* < 0.05, Table 4), while it did not affect the BWG and FCR (*P* > 0.05, Table 4). BA supplementation did not significantly alter ADFI, BWG, or FCR during this period (*P* > 0.05, Table 4). Production performance from d 22 to 42 showed that under LFD, BAs significantly increased both ADFI and BWG of broiler chickens (*P* < 0.05, Table 4). The HFD resulted in a significant increase in BWG during this period (*P* < 0.05, Table 4), with no significant impact on ADFI or FCR (*P* > 0.05, Table 4). Analysis of production performance from d 1 to 42 showed that the HFD significantly elevated both ADFI and BWG during this period (*P* < 0.05, Table 4), with no significant effect on FCR (*P* > 0.05, Table 4). BAs exhibited a trend of increasing ADFI from d 1 to 42 (0.05 < *P* < 0.1, Table 4), while they did not significantly affect BWG or FCR during this period (*P* > 0.05, Table 4). Notably, there was no interaction between the effects of the HFD and BAs on the production performance of broiler chickens from d 1 to 42 (*P* > 0.05, Table 4).

**Table 4:** Effects of dietary bile acids supplementation (BAs, 60 mg/kg) and high-fat diet (HFD) on the production performance of broiler chickens

Parameters	BAs	Diet			SEM	*P* values
		LFD	HFD	Means		
1–21 d						
ADFI, g/d	Control	39.45	42.24	40.85	1.04	Diet: 0.013
	BAs	37.08	42.10	39.59	1.04	BAs: 0.401
	Means	38.27^b^	42.17^a^		1.47	Diet × BAs: 0.455
BWG, g/d	Control	28.25	29.59	28.92	0.63	Diet: 0.084
	BAs	28.22	30.05	29.14	0.63	BAs: 0.718
	Means	28.24	29.82		0.88	Diet × BAs: 0.785
FCR, g/g	Control	1.40	1.55	1.48	0.06	Diet: 0.238
	BAs	1.37	1.41	1.39	0.06	BAs: 0.264
	Means	1.39	1.48		0.08	Diet × BAs: 0.492
22–42 d						
ADFI, g/d	Control	121.5	138.7	130.1	5.32	Diet: 0.094
	BAs	139.8	148.6	144.2	5.32	BAs: 0.071
	Means	130.7	143.7		7.52	Diet × BAs: 0.580
BWG, g/d	Control	63.99	73.48	68.92	1.77	Diet: 0.011
	BAs	71.00	74.85	72.93	1.77	BAs: 0.127
	Means	67.50^b^	74.17^a^		2.42	Diet × BAs: 0.270
FCR, g/g	Control	1.90	1.90	1.90	0.07	Diet: 0.483
	BAs	1.91	2.11	2.01	0.07	BAs: 0.147
	Means	1.91	2.01		0.10	Diet × BAs: 0.473
1–42 d						
ADFI, g/d	Control	80.46	90.48	85.47	2.38	Diet: 0.018
	BAs	88.44	95.37	91.91	2.38	BAs: 0.066
	Means	84.45^b^	92.93^a^		3.36	Diet × BAs: 0.646
BWG, g/d	Control	46.12	50.72	48.42	1.09	Diet: 0.057
	BAs	49.25	50.75	50.00	1.09	BAs: 0.312
	Means	47.69	50.74		1.53	Diet × BAs: 0.320
FCR, g/g	Control	1.75	1.79	1.77	0.04	Diet: 0.280
	BAs	1.80	1.89	1.85	0.04	BAs: 0.193
	Means	1.78	1.84		0.06	Diet × BAs: 0.663

Data are presented as the mean ± SEM (*n* = 8)

*LFD* Low-fat diet with 2.22% and 4.79% EE, *HFD* High-fat diet with 5.40% and 7.65% EE

^a,b^Means with different superscript within diet treatment differ significantly (*P* < 0.05)

### Effects of BAs on organ indices in broiler chickens fed diets with different fat levels

Organ indices of broiler chickens at 21 days of age revealed that under the condition of adding BAs to both diets, the HFD significantly increased the liver weight, decreased gallbladder weight, gallbladder index, and pancreas index, increased duodenal weight, ileal weight, and ileum index while decreasing jejunal weight in broiler chickens at 21 days of age (*P* < 0.05, Table 5). The HFD also significantly reduced the liver index, pancreas index, and increased ileal weight and ileum index (*P* < 0.05, Table 5). BA supplementation notably increased the leg muscle index (*P* < 0.05, Table 5) but did not significantly affect other indices (*P* > 0.05, Table 5). Moreover, there was no significant interaction between the effects of the HFD and BAs on the organ indices of broiler chickens at 21 days of age (*P* > 0.05, Table 5). Organ indices at 42 days of age showed that BA treatment significantly affected gallbladder index and pancreas index (*P* < 0.05, Table 6). Additionally, a significant interaction was observed between the effects of the HFD and BAs on pancreas weight (*P* < 0.05, Table 6).

**Table 5:** Effects of dietary bile acids supplementation (BAs, 60 mg/kg) and high-fat diet (HFD) on organ indices of broiler chickens on 21 days of age

Parameters	BAs	Diet			SEM	*P* values
		LFD	HFD	Means		
Liver						
Weight, g	Control	18.94	21.24	20.09	1.04	Diet: 0.022
	BAs	17.17	19.94	18.56	1.04	BAs: 0.152
	Means	18.06^b^	20.59^a^		1.47	Diet × BAs: 0.822
Index, %	Control	2.76	2.40	2.58	0.11	Diet: 0.157
	BAs	2.50	2.51	2.50	0.11	BAs:0.516
	Means	2.63	2.46		0.16	Diet × BAs: 0.120
Gall bladder						
Weight, g	Control	0.59	0.77	0.68	0.13	Diet: 0.551
	BAs	0.46	0.43	0.44	0.13	BAs: 0.082
	Means	0.52	0.60		0.18	Diet × BAs: 0.418
Index, %	Control	0.08	0.11	0.10	0.02	Diet: 0.759
	BAs	0.07	0.06	0.06	0.02	BAs: 0.119
	Means	0.08	0.08		0.03	Diet × BAs: 0.352
Breast muscle						
Weight, g	Control	116.1	124.1	120.1	10.00	Diet: 0.445
	BAs	118.7	126.3	122.5	10.00	BAs: 0.810
	Means	117.4	125.2		14.07	Diet × BAs: 0.984
Index, %	Control	16.50	17.26	16.88	0.90	Diet: 0.781
	BAs	17.00	16.75	16.88	0.90	BAs: 0.996
	Means	16.75	17.01		1.28	Diet × BAs: 0.579
Thigh muscle						
Weight, g	Control	93.11	93.71	93.41	5.52	Diet: 0.872
	BAs	101.1	102.3	101.7	5.52	BAs: 0.148
	Means	97.11	98.01		7.80	Diet × BAs: 0.957
Index, %	Control	13.30	13.08	13.19^y^	0.38	Diet: 0.153
	BAs	14.57	13.68	14.13^x^	0.38	BAs: 0.020
	Means	13.94	13.38		0.54	Diet × BAs: 0.374
Abdominal fat						
Weight, g	Control	6.49	5.54	6.01	1.11	Diet: 0.805
	BAs	4.91	5.30	5.11	1.11	BAs: 0.423
	Means	5.70	5.42		1.58	Diet × BAs: 0.557
Index, %	Control	0.93	0.74	0.84	0.15	Diet: 0.536
	BAs	0.71	0.71	0.71	0.15	BAs: 0.401
	Means	0.82	0.73		0.21	Diet × BAs: 0.529
Thymus						
Weight, g	Control	1.87	2.41	2.14	0.30	Diet: 0.124
	BAs	2.01	2.44	2.23	0.30	BAs: 0.781
	Means	1.94	2.43		0.43	Diet × BAs: 0.853
Index, %	Control	0.27	0.35	0.31	0.04	Diet: 0.164
	BAs	0.29	0.33	0.31	0.04	BAs: 0.977
	Means	0.28	0.34		0.06	Diet × BAs: 0.640
Spleen						
Weight, g	Control	0.77	0.86	0.81	0.15	Diet: 0.961
	BAs	0.91	0.84	0.88	0.15	BAs: 0.664
	Means	0.84	0.85		0.21	Diet × BAs: 0.595
Index, %	Control	0.27	0.35	0.31	0.04	Diet: 0.164
	BAs	0.29	0.33	0.31	0.04	BAs: 0.977
	Means	0.28	0.34		0.06	Diet × BAs: 0.640
Bursa						
Weight, g	Control	1.77	1.46	1.61	0.15	Diet: 0.021
	BAs	1.70	1.29	1.49	0.15	BAs: 0.412
	Means	1.74^a^	1.37^b^		0.21	Diet × BAs: 0.738
Index, %	Control	0.26	0.20	0.23	0.02	Diet: 0.003
	BAs	0.24	0.17	0.21	0.02	BAs: 0.284
	Means	0.25^a^	0.19^b^		0.03	Diet × BAs: 0.711
Pancreas						
Weight, g	Control	2.79	2.33	2.56	0.20	Diet: 0.040
	BAs	2.66	2.24	2.45	0.20	BAs: 0.592
	Means	2.72^a^	2.28^b^		0.30	Diet × BAs: 0.921
Index, %	Control	0.40	0.32	0.36	0.03	Diet: 0.007
	BAs	0.38	0.31	0.35	0.03	BAs: 0.476
	Means	0.39^a^	0.32^b^		0.04	Diet × BAs: 0.947
Duodenum						
Weight, g	Control	7.01	7.91	7.46	0.39	Diet: 0.031
	BAs	6.44	7.31	6.88	0.39	BAs: 0.142
	Means	6.73^b^	7.61^a^		0.55	Diet × BAs: 0.971
Index, %	Control	1.02	1.11	1.07	0.05	Diet: 0.192
	BAs	0.93	0.98	0.96	0.05	BAs: 0.058
	Means	0.97	1.05		0.08	Diet × BAs: 0.731
Jejunum						
Weight, g	Control	9.84	9.07	9.46	0.59	Diet: 0.428
	BAs	10.56	10.37	10.46	0.59	BAs: 0.103
	Means	10.20	9.72		0.84	Diet × BAs: 0.626
Index, %	Control	1.42	1.51	1.47	0.10	Diet: 0.452
	BAs	1.32	1.39	1.35	0.10	BAs: 0.276
	Means	1.37	1.45		0.14	Diet × BAs: 0.923
Ileum						
Weight, g	Control	15.17	18.79	16.98	1.08	Diet: 0.001
	BAs	14.40	19.19	16.79	1.08	BAs: 0.865
	Means	14.79^b^	18.99^a^		1.52	Diet × BAs: 0.592
Index, %	Control	2.20	2.66	2.43	0.18	Diet: 0.015
	BAs	2.10	2.60	2.35	0.18	BAs: 0.672
	Means	2.15^b^	2.63^a^		0.26	Diet × BAs: 0.912
Cecum						
Weight, g	Control	4.80	5.84	5.32	0.51	Diet: 0.195
	BAs	4.79	5.11	4.95	0.51	BAs: 0.477
	Means	4.79	5.48		0.73	Diet × BAs: 0.494
Index, %	Control	0.69	0.84	0.76	0.08	Diet: 0.398
	BAs	0.69	0.69	0.69	0.08	BAs: 0.341
	Means	0.69	0.76		0.11	Diet × BAs: 0.363

Data are presented as the mean ± SEM (*n* = 8)

*LFD* Low-fat diet with 2.22% and 4.79% EE, *HFD* High-fat diet with 5.40% and 7.65% EE

^a,b^Means with different superscript letters within diet treatment differ significantly (*P* < 0.05)

^x,y^Means with different superscript letters within BAs treatment differ significantly (*P* < 0.05)

**Table 6:** Effects of dietary bile acids supplementation (BAs, 60 mg/kg) and high-fat diet (HFD) on organ indices of broiler chickens on 42 days of age

Parameters	BAs	Diet			SEM	*P* values
		LFD	HFD	Means		
Liver						
Weight, g	Control	35.04	34.44	34.74	1.86	Diet: 0.758
	BAs	37.33	36.29	36.81	1.86	BAs: 0.441
	Means	36.19	35.37		2.63	Diet × BAs: 0.934
Index, %	Control	1.99	1.87	1.93	0.07	Diet: 0.692
	BAs	1.86	1.90	1.88	0.07	BAs: 0.804
	Means	1.43	1.89		0.10	Diet × BAs: 0.316
Gall bladder						
Weight, g	Control	1.32	1.07	1.20	0.11	Diet: 0.261
	BAs	1.28	1.16	1.22	0.11	BAs: 0.850
	Means	1.30	1.12		0.16	Diet × BAs: 0.715
Index, %	Control	0.07	0.05	0.06	0.01	Diet: 0.067
	BAs	0.08	0.06	0.07	0.01	BAs: 0.327
	Means	0.08	0.06		0.01	Diet × BAs: 0.609
Breast muscle						
Weight, g	Control	322.8	341.8	332.3	16.89	Diet: 0.662
	BAs	376.6	336.4	356.5	16.89	BAs: 0.321
	Means	349.7	339.1		23.89	Diet × BAs: 0.227
Index, %	Control	18.05	18.18	18.12	0.35	Diet: 0.606
	BAs	17.71	18.06	17.89	0.35	BAs: 0.593
	Means	17.88	18.12		0.49	Diet × BAs: 0.437
Thigh muscle						
Weight, g	Control	271.6	284.7	278.2	13.60	Diet: 0.498
	BAs	326.2	286.7	306.5	13.60	BAs: 0.155
	Means	298.9	285.7		19.24	Diet × BAs: 0.185
Index, %	Control	15.33	15.13	15.23	0.24	Diet: 0.151
	BAs	16.17	15.35	15.76	0.24	BAs: 0.142
	Means	15.75	15.24		0.35	Diet × BAs: 0.374
Abdominal fat						
Weight, g	Control	25.54	26.31	25.93	2.90	Diet: 0.522
	BAs	18.78	21.77	20.28	2.90	BAs: 0.063
	Means	21.86	24.04		4.10	Diet × BAs: 0.705
Index, %	Control	1.37	1.47	1.42^x^	0.12	Diet: 0.517
	BAs	1.00	1.07	1.04^y^	0.12	BAs: 0.005
	Means	1.19	1.27		0.18	Diet × BAs: 0.916
Thymus						
Weight, g	Control	4.26	5.07	4.67	0.59	Diet: 0.375
	BAs	4.46	5.14	4.80	0.59	BAs: 0.871
	Means	4.36	5.11		0.83	Diet × BAs: 0.939
Index, %	Control	0.23	0.26	0.25	0.03	Diet: 0.314
	BAs	0.22	0.27	0.25	0.03	BAs: 0.987
	Means	0.23	0.27		0.04	Diet × BAs: 0.780
Spleen						
Weight, g	Control	2.30	2.25	2.28	0.16	Diet: 0.831
	BAs	2.57	2.52	2.55	0.16	BAs: 0.244
	Means	2.44	2.39		0.22	Diet × BAs: 0.994
Index, %	Control	0.13	0.13	0.13	0.01	Diet: 0.313
	BAs	0.12	0.15	0.14	0.01	BAs:0.722
	Means	0.13	0.14		0.01	Diet × BAs: 0.288
Bursa						
Weight, g	Control	3.40	3.93	3.67	0.23	Diet: 0.865
	BAs	3.76	3.19	3.48	0.23	BAs: 0.567
	Means	3.58	3.56		0.33	Diet × BAs: 0.115
Index, %	Control	0.20	0.21	0.21^x^	0.01	Diet: 0.778
	BAs	0.17	0.17	0.17^y^	0.01	BAs: 0.015
	Means	0.19	0.19		0.01	Diet × BAs: 0.673
Pancreas						
Weight, g	Control	4.11^m^	4.43^m^	4.27	0.13	Diet: 0.462
	BAs	4.23^m^	3.67^n^	3.94	0.13	BAs: 0.101
	Means	4.17	4.05		0.18	Diet × BAs: 0.025
Index, %	Control	0.23	0.23	0.23^x^	0.01	Diet: 0.283
	BAs	0.21	0.20	0.21^y^	0.01	BAs: 0.023
	Means	0.22	0.22		0.01	Diet × BAs: 0.832
Duodenum						
Weight, g	Control	8.40	9.73	9.07	0.42	Diet: 0.314
	BAs	9.33	9.28	9.31	0.42	BAs: 0.723
	Means	8.87	9.51		0.60	Diet × BAs: 0.243
Index, %	Control	0.48	0.52	0.50	0.02	Diet: 0.244
	BAs	0.44	0.49	0.47	0.02	BAs: 0.507
	Means	0.46	0.51		0.03	Diet × BAs: 0.675
Jejunum						
Weight, g	Control	18.14	18.96	18.55	0.91	Diet: 0.617
	BAs	18.97	19.46	10.72	0.91	BAs: 0.609
	Means	18.56	19.21	14.64	1.28	Diet × BAs: 0.899
Index, %	Control	1.03	1.01	1.02	0.04	Diet: 0.685
	BAs	0.97	1.03	1.00	0.04	BAs: 0.783
	Means	1.00	1.02		0.06	Diet × BAs: 0.535
Ileum						
Weight, g	Control	27.16	34.40	30.78	1.88	Diet: 0.278
	BAs	29.39	28.07	28.73	1.88	BAs: 0.450
	Means	28.28	31.24		2.67	Diet × BAs: 0.121
Index, %	Control	1.51	1.81	1.66	0.07	Diet: 0.122
	BAs	1.48	1.51	1.50	0.07	BAs: 0.130
	Means	1.50	1.66		0.10	Diet × BAs: 0.210
Cecum						
Weight, g	Control	6.89	6.33	6.61	0.43	Diet: 0.782
	BAs	6.73	6.94	6.84	0.43	BAs: 0.713
	Means	6.81	6.64		0.61	Diet × BAs: 0.536
Index, %	Control	0.39	0.34	0.37	0.02	Diet: 0.733
	BAs	0.34	0.37	0.36	0.02	BAs: 0.768
	Means	0.37	0.36		0.03	Diet × BAs: 0.157

Data are presented as the mean ± SEM (*n* = 8)

*LFD* Low-fat diet with 2.22% and 4.79% EE, *HFD* High-fat diet with 5.40% and 7.65% EE

^m,n^Means with different superscript letters differ significantly (*P* < 0.05)

^x,y^Means with different superscript letters within BAs treatment differ significantly (*P* < 0.05)

### Effects of BAs on plasma biochemical parameters in broiler chickens fed diets with different fat levels

**Fig. 1:**
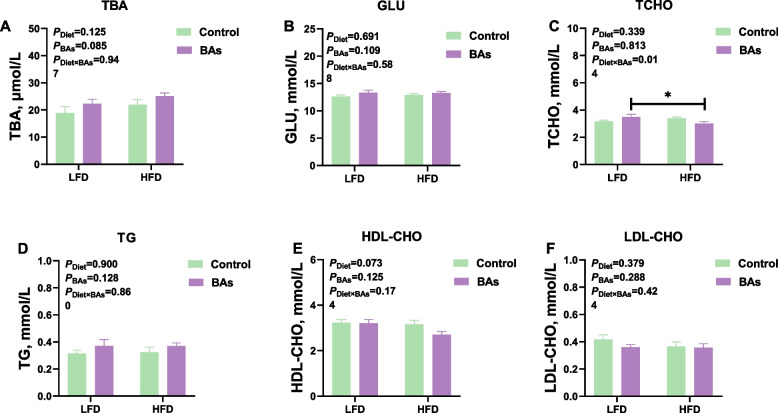
Effects of BAs on plasma biochemical parameters in 21-day-old broiler chickens fed diets with different fat levels. **A** TBA content; **B** GLU content; **C** TCHO content; **D** TG content; **E** HDL-CHO content; **F** LDL-CHO content. Data are presented as the mean ± SEM (*n* = 8). The *P* values in the top left corner of each panel represent the results of two-way ANOVA. *P*_Diet_ < 0.05 indicates a significant effect of dietary fat level on the respective parameter, *P*_BAs_ < 0.05 indicates a significant effect of BAs on the respective parameter, and *P*_Diet×BAs_ < 0.05 indicates a significant interaction effect between dietary fat level and BAs on the respective parameter. ^*^P < 0.05

**Fig. 2:**
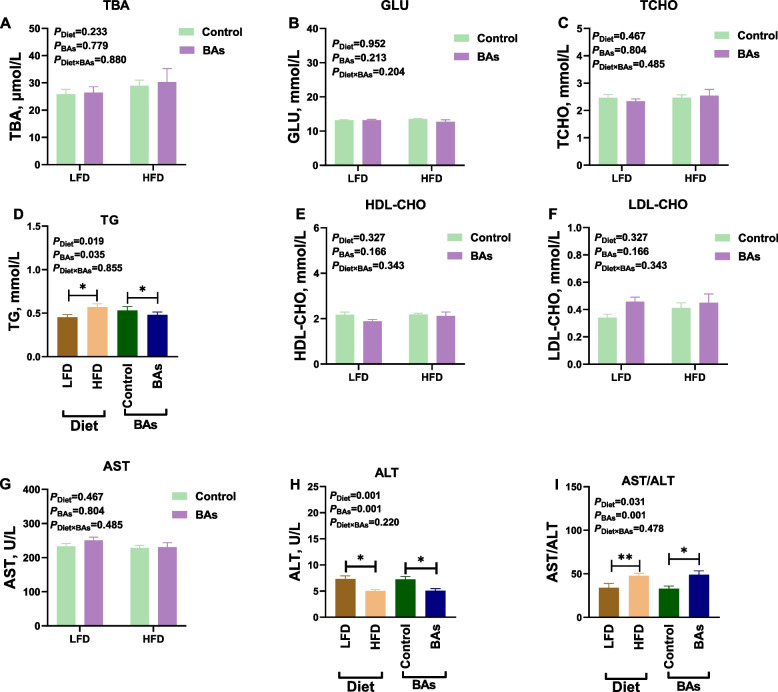
Effects of BAs on plasma biochemical parameters in 42-day-old broiler chickens fed diets with different fat levels. **A** TBA content; **B** GLU content; **C** TCHO content; **D** TG content; **E** HDL-CHO content; **F** LDL-CHO content; **G** AST content; **H** ALT content; **I** AST/ALT ratio. Data are presented as the mean ± SEM (*n* = 8). The *P* values in the top left corner of each panel represent the results of two-way ANOVA. *P*_Diet_ < 0.05 indicates a significant effect of dietary fat level on the respective parameter, *P*_BAs_ < 0.05 indicates a significant effect of BAs on the respective parameter, and *P*_Diet×BAs_ < 0.05 indicates a significant interaction effect between dietary fat level and BAs on the respective parameter. ^*^P < 0.05, ^**^P < 0.01

Analysis of plasma biochemical parameters related to lipid metabolism in broiler chickens at 21 days of age showed that neither the HFD nor the BAs treatment had a significant effect on the plasma levels of TBA, GLU, TCHO, TG, HDL-CHO, and LDL-CHO (*P* > 0.05, Fig. 1A–F). However, the results in Fig. 1C shows a significant interaction between BAs and HFD treatments on the plasma TCHO levels in 21-day-old broiler chickens (*P* < 0.05, Fig. 1C). Analysis of these parameters in the plasma of broiler chickens at 42 days of age showed that neither the HFD nor the BA treatment significantly affected the levels of TBA, GLU, TCHO, HDL-CHO, LDL-CHO, and AST in plasma (*P* > 0.05, Fig. 2A–G). However, the BAs treatment significantly reduced the plasma TG levels and ALT enzyme activity in broiler chickens fed both LFD and HFD (*P* < 0.05, Fig. 2D and H), while the HFD treatment significantly increased plasma TG levels and decreased ALT enzyme activity (*P* < 0.05, Fig. 2D and H). Both the HFD and BAs treatments increased the AST/ALT ratio in the plasma of broiler chickens at 42 days of age (*P* < 0.05, Fig. 2I).

### Effects of BAs on hepatic lipid metabolism in broiler chickens fed diets with different fat levels

**Fig. 3:**
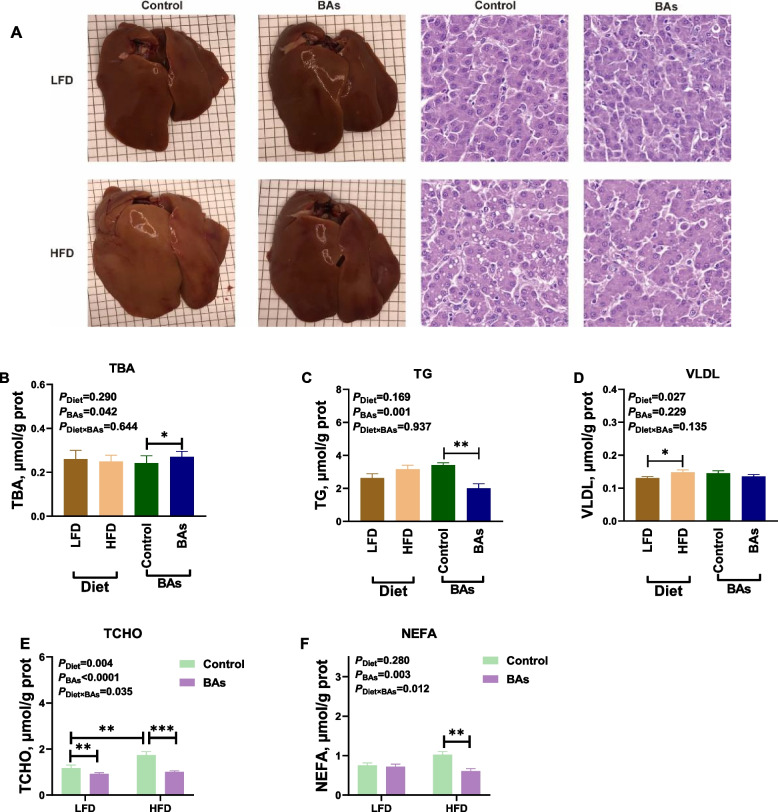
Effects of BAs on hepatic lipid metabolism in 42-day-old broiler chickens fed diets with different fat levels. **A** Hepatic tissue morphology and HE staining; **B** Hepatic tissue TBA content; **C** Hepatic tissue TG content; **D** Hepatic tissue VLDL content; **E** Hepatic tissue TCHO content; **F** Hepatic tissue NEFA content. Data are presented as the mean ± SEM (*n* = 8). The *P* values in the top left corner of each panel represent the results of two-way ANOVA. *P*_Diet_ < 0.05 indicates a significant effect of dietary fat level on the respective parameter, *P*_BAs_ < 0.05 indicates a significant effect of BAs on the respective parameter, and *P*_Diet×BAs_ < 0.05 indicates a significant interaction effect between dietary fat level and BAs on the respective parameter. ^*^P < 0.05, ^**^P < 0.01, ^***^P < 0.001

Analysis of hepatic lipid metabolism-related parameters in 42-day-old broiler chickens showed that feeding an HFD diet led to a yellowish liver color, and histological examination using HE staining presented the presence of numerous lipid droplets in the liver tissues of HFD-fed chickens (Fig. 3A). However, supplementation of BAs to the HFD regimen showed alleviated lipid droplets in the liver (Fig. 3A). BAs treatment significantly increased the content of TBA in the liver tissues of broiler chickens while decreasing the levels of TG, TCHO, and NEFA (*P* < 0.05, Fig. 3C, E and F). BA treatment had no significant effect on VLDL content in the liver of broiler chickens (*P* > 0.05, Fig. 3D). Different from BAs, HFD treatment significantly increased the levels of VLDL and TCHO in the liver tissues of broiler chickens (*P* < 0.05, Fig. 3E). Additionally, there was a significant interaction effect between the HFD and BAs on the levels of TCHO and NEFA in the liver tissues of broiler chickens (*P* < 0.05, Fig. 3E and F).

**Fig. 4:**
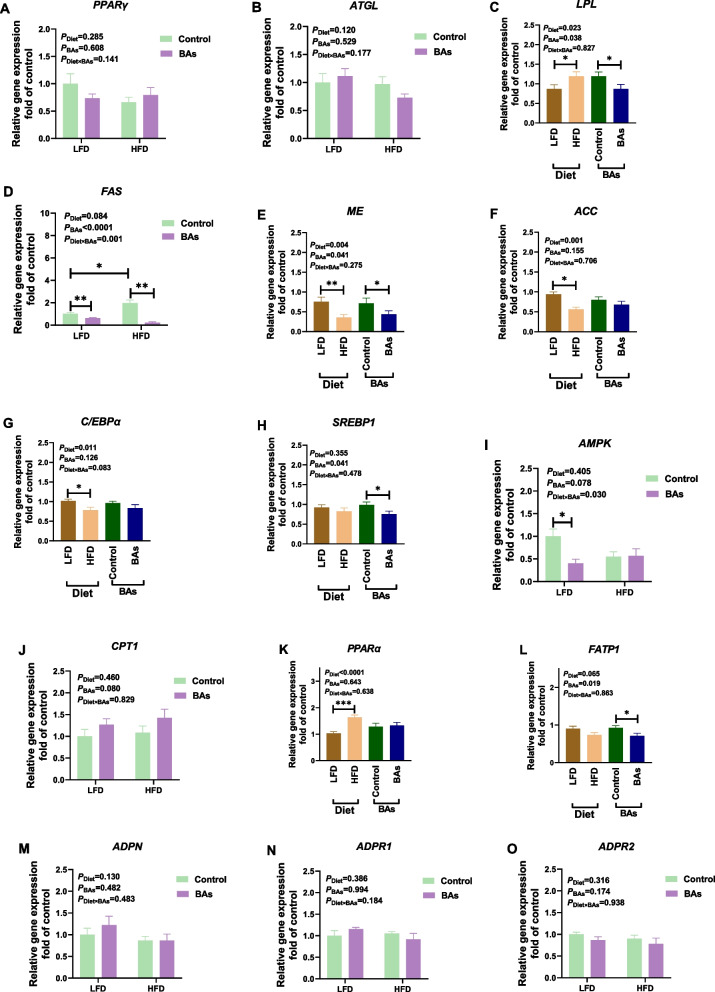
Effects of BAs on mRNA levels of hepatic lipid metabolism-related genes in 42-day-old broiler chickens fed diets with different fat levels. **A** *PPARγ*; **B** *ATGL*; **C** *LPL*; **D** *FAS*; **E** *ME*; **F** *ACC*; **G** *C/EPBα*; **H** *SREBP1*; **I** *AMPK*; **J** *CPT1*; **K** *PPARα*; **L** *FATP1*; **M** *ADPN*; **N** *ADPR1*; **O** *ADPR2*. Data are presented as the mean ± SEM (*n* = 8). The *P* values in the top left corner of each panel represent the results of two-way ANOVA. *P*_Diet_ < 0.05 indicates a significant effect of dietary fat level on the respective parameter, *P*_BAs_ < 0.05 indicates a significant effect of BAs on the respective parameter, and *P*_Diet×BAs_ < 0.05 indicates a significant interaction effect between dietary fat level and BAs on the respective parameter. ^*^P < 0.05, ^**^P < 0.01

Analysis of gene expression levels related to hepatic lipid metabolism in 42-day-old broiler chickens revealed that HFD treatment significantly upregulated the expressions of *LPL*, *FAS*, and *PPARα* in the liver tissues (*P* < 0.05, Fig. 4C, D, and K). HFD treatment also significantly downregulated the expressions of *ME*, *ACC*, *C/EBPα*, and *AMPK* in the liver tissues (*P* < 0.05, Fig. 4E–G, I). BA treatment significantly downregulated the expressions of *LPL*, *FAS*, *ME*, *SREBP1*, and *FATP1* in the liver tissues (*P* < 0.05, Fig. 4C–E, H, and L). Furthermore, there was a significant interaction effect of HFD and BAs on the expressions of *FAS* and *AMPK* in the liver tissues (*P* < 0.05, Fig. 4D and I). The expressions of *PPARγ*, *ATGL*, *CPT1*, *ADPN*, *ADPR1*, and *ADPR2* in the liver tissues of broiler chickens were not significantly affected by HFD or BAs treatment (*P* > 0.05, Fig. 4A, B, J, and M–O).

### Effects of BAs on hepatic BA composition in broiler chickens fed diets with different fat levels

**Fig. 5:**
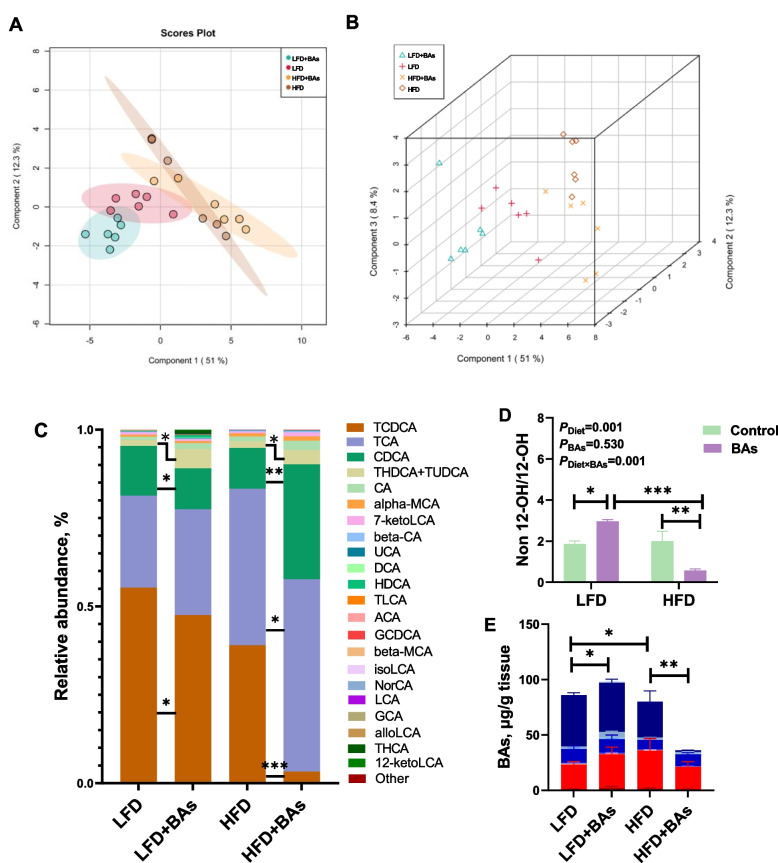
Effects of BAs on hepatic BA composition in broiler chickens fed diets with different fat levels. **A** PCA 2D Analysis; **B** PCA 3D Analysis; **C** Composition of BAs; **D** Ratio of Non 12-OH/12-OH BAs; **E** Content of Non 12-OH BAs and 12-OH BAs. Data are presented as the mean ± SEM (*n* = 8). ^*^P < 0.05, ^**^P < 0.01, ^***^P < 0.001. **E** displays the color coding used for BA types. The red color indicates 12-OH BAs, while the blue color represents Non 12-OH BAs. Specifically, 12-OH BAs consist of TCA, CA, NorCA, DCA, beta-CA, and ACA. Non 12-OH BAs encompass TCDCA, CDCA, alloLCA, LCA, isoLCA, 7-ketoLCA, HDCA, alpha-MCA, beta-MCA, UCA, GHDCA, GCDCA, GUDCA, TLCA, and THDCA + TUDCA. The asterisk (*) in Panel **E** highlights the disparity in Non 12-OH BA content between the compared groups

Analysis of hepatic BA content in broiler chicken tissues presented obvious variations in the composition and types of BAs among the four groups, as indicated by PCA 2D and 3D analyses (Fig. 5A and B). This suggests that the four diets have a substantial impact on the composition and structure of BAs in liver tissues of broiler chicken. Examination of BA composition and proportions in the four groups showed that compared to the LFD group, adding BAs to the LFD decreased the proportions of taurochenodeoxycholic acid (TCDCA) and chenodeoxycholic acid (CDCA) in liver tissue while increasing the proportions of taurolithocholic acid (THDCA) and tauroursodeoxycholic acid (TUDCA) (*P* < 0.05, Fig. 5C). Similarly, compared to the LFD group, the HFD group exhibited decreased proportions of TCDCA and CDCA in liver tissue and an increased proportion of taurocholic acid (TCA) (*P* < 0.05, Fig. 5C). Moreover, compared to the HFD group, adding BAs to the HFD diet significantly reduced the proportion of TCDCA in liver tissue while increasing the proportions of TCA, CDCA, and THDCA + TUDCA (*P* < 0.05, Fig. 5C). Analysis of the absolute contents of 12-hydroxylated BAs (12-OH BAs) and Non-12-hydroxylated BAs (Non 12-OH BAs) in the four groups showed that relative to LFD group, the LFD + BAs group had a significantly higher content of Non 12-OH BAs in liver tissue (*P* < 0.05, Fig. 5E). Conversely, compared to the LFD group, the HFD group exhibited a significantly lower content of Non 12-OH BAs in liver tissue (*P* < 0.05, Fig. 5E). Contrary to the LFD, the HFD + BAs group had a significantly lower content of Non 12-OH BAs in liver tissue than HFD group (*P* < 0.05, Fig. 5E). Analysis of the Non 12-OH/12-OH BAs ratio in the four groups showed that relative to the LFD group, the LFD + BAs group exhibited a significantly higher Non 12-OH/12-OH BAs ratio (*P* < 0.05, Fig. 5D). Conversely, relative to the HFD group, the HFD + BAs group showed a significantly lower Non 12-OH/12-OH BAs ratio (*P* < 0.05, Fig. 5D). Additionally, compared to the LFD + BAs group, the HFD + BAs group displayed a significantly lower Non 12-OH/12-OH BAs ratio (*P* < 0.05, Fig. 5D).

### Effects of BAs on the synthesis and transport of BAs in broiler chickens fed diets with different fat levels

**Fig. 6:**
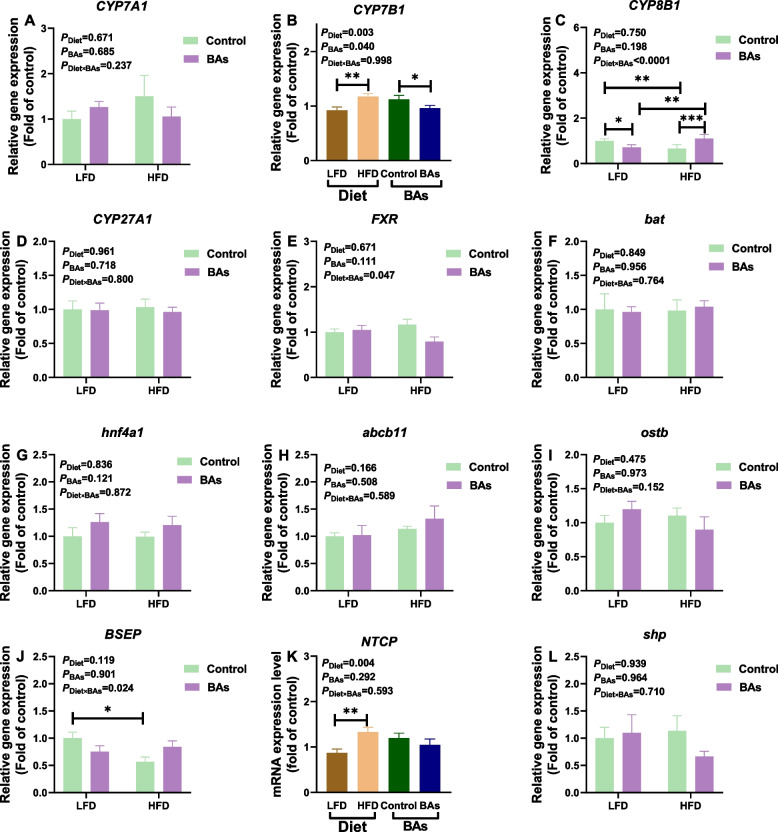
Effects of BAs on the synthesis and transport of bas in broiler chickens fed diets with different fat levels. **A** *CYP7A1*; **B** *CYP7B1*; **C** *CYP8B1*; **D** *CYP27A1*; **E** *FXR*; **F** *bat*; **G** *hnf4a1*; **H** *abcb11*; **I** *ostb*; **J** *BSEP*; **K** *NTCP*; and **L** *shp*. Data are presented as the mean ± SEM (*n* = 8). The *P* values in the top left corner of each panel represent the results of two-way ANOVA. *P*_Diet_ < 0.05 indicates a significant effect of dietary fat level on the respective parameter, *P*_BAs_ < 0.05 indicates a significant effect of BAs on the respective parameter, and *P*_Diet×BAs_ < 0.05 indicates a significant interaction effect between dietary fat level and BAs on the respective parameter. ^*^P < 0.05, ^**^P < 0.01, ^***^P < 0.001

Additionally, we analyzed the mRNA levels of enzymes associated with BA synthesis and transport in liver tissues. The results showed that both BAs and HFD treatments had no significant effect on the expressions of *CYP7A1*, *CYP27A1*, *FXR*, *bat*, *hnf4a1*, *abcb11*, *ostb*, and *shp* in broiler chicken liver tissues (*P* > 0.05, Fig. 6A, D–I, L). Compared to the LFD group, the HFD group exhibited significant increased expression levels of *CYP7B1* and *NTCP* (*P* < 0.05, Fig. 6B and K) and decreased expression levels of *CYP8B1* and *BSEP* (*P* < 0.05, Fig. 6C and J). Relative to the Control group, BAs significantly reduced the expression of *CYP7B1* and *CYP8B1* in LFD + BAs group (*P* < 0.05, Fig. 6B and C). Furthermore, compared to the HFD group, the HFD + BAs group also showed a significant increase in the expression of *CYP8B1* (*P* < 0.05, Fig. 6C). There was a significant interaction effect of HFD and BAs on the expressions of *CYP8B1* and *BSEP* in liver tissues of broiler chickens (*P* < 0.05, Fig. 6C and J).

### Effects of BAs on gut microbiota in broiler chickens fed diets with different fat levels

**Fig. 7:**
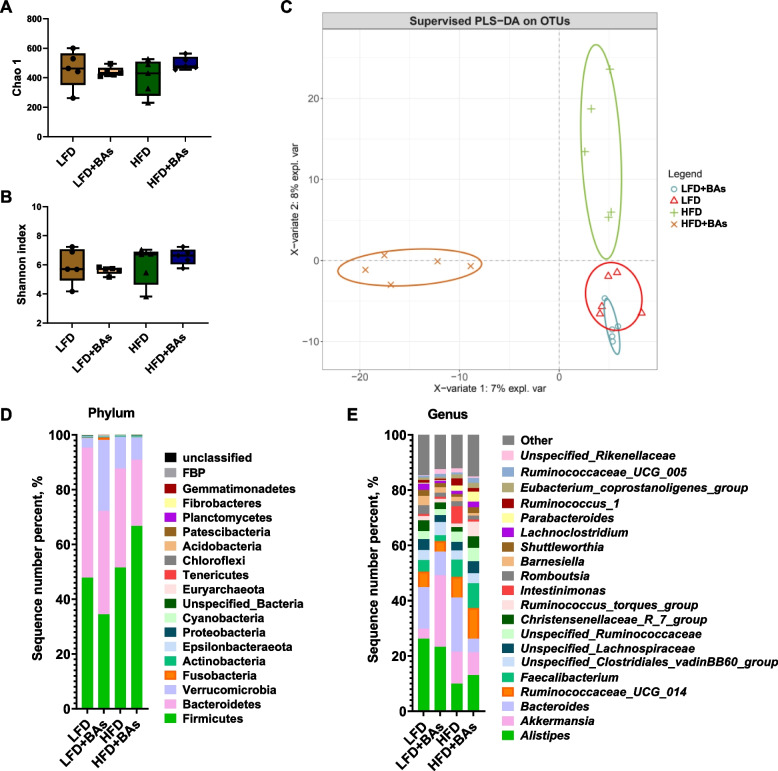
Effects of BAs on the cecal microbiota in broiler chickens fed diets with different fat levels. **A** Chao 1 analysis; **B** Shannon index; **C** PCoA; **D** The distribution of cecum microbiota at the phylum level; **E** The distribution of cecum microbiota at the genus level. *n* = 6

**Fig. 8:**
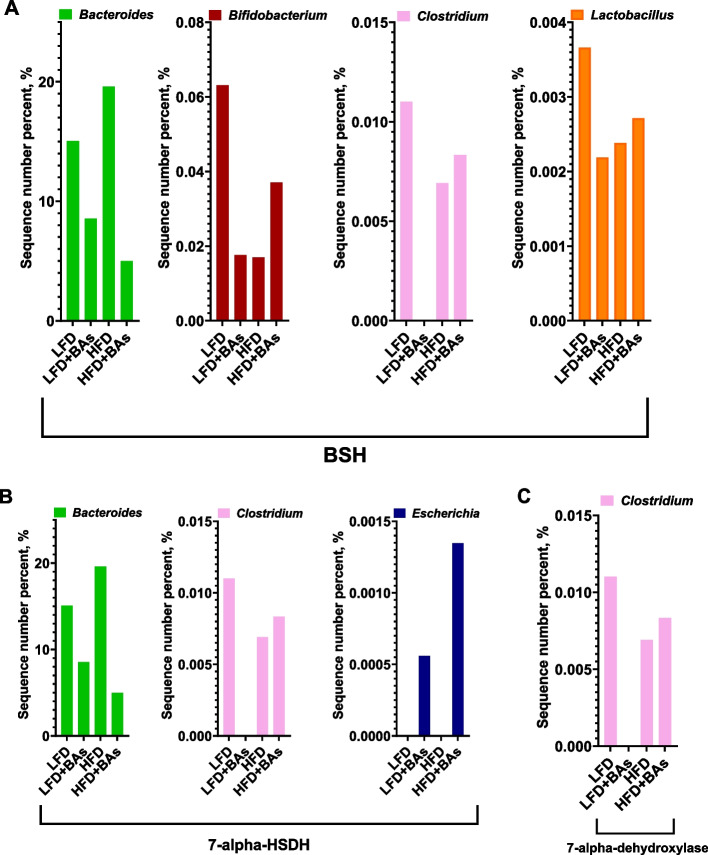
Effects of BAs on the strains encoded by BA-metabolizing enzymes in broiler chickens fed diets with different fat levels. **A** The average abundance of bacterial genera expressing BSH. **B** The average abundance of bacterial genera expressing 7-alpha HSDH. **C** The average abundance of bacterial genera expressing 7-alpha-dehydroxylase (*Clostridium*). *n* = 6

Analysis of the gut microbiota in broiler chickens showed that adding BAs to both LFD and HFD diets had no significant effect on the Chao 1 and Shannon indices of the cecum microbiota (*P* > 0.05, Fig. 7A and B). Partial Least Squares Discriminant Analysis (PLS-DA) showed a high degree of dispersion in the cecum microbiota of chickens from all four groups, indicating that HFD and BA treatment influenced the structure and composition of the gut microbiota in broiler chickens (Fig. 7C). Analysis of the microbial structure and composition at the phylum and genus levels showed that at the phylum level, compared with the LFD group, the LFD + BAs group had a decreased abundance of Firmicutes and an increased abundance of Verrucomicrobia in the cecum microbiota (Fig. 7D). Furthermore, the HFD + BAs group exhibited an increased abundance of Firmicutes and decreased abundance of Bacteroidetes and Verrucomicrobia in the cecum microbiota compared to the HFD group (Fig. 7D). Analysis of the gut microbiota at the genus level showed that compared with the LFD group, the LFD + BAs group had decreased abundances of *Alistipes* and *Bacteroides* and an increased abundance of *Akkermansia* in the cecum microbiota (Fig. 7E). Additionally, analysis of bacterial encoding bile acid hydrolases in the cecum microbiota revealed that both LFD and HFD supplemented with BAs reduced the abundance of *Bacteroides* and increased the abundance of *Escherichia* in the cecum microbiota of broiler chickens (Fig. 8A, B). However, BA supplementation in LFD reduced the abundances of *Bifidobacterium*, *Clostridium*, and *Lactobacillus* in cecum microbiota of broiler chickens, while in the HFD, it increased the abundance of these genera (Fig. 8A–C).

### Correlation analysis of BA composition in the liver and gut microbiota of broiler chickens

**Fig. 9:**
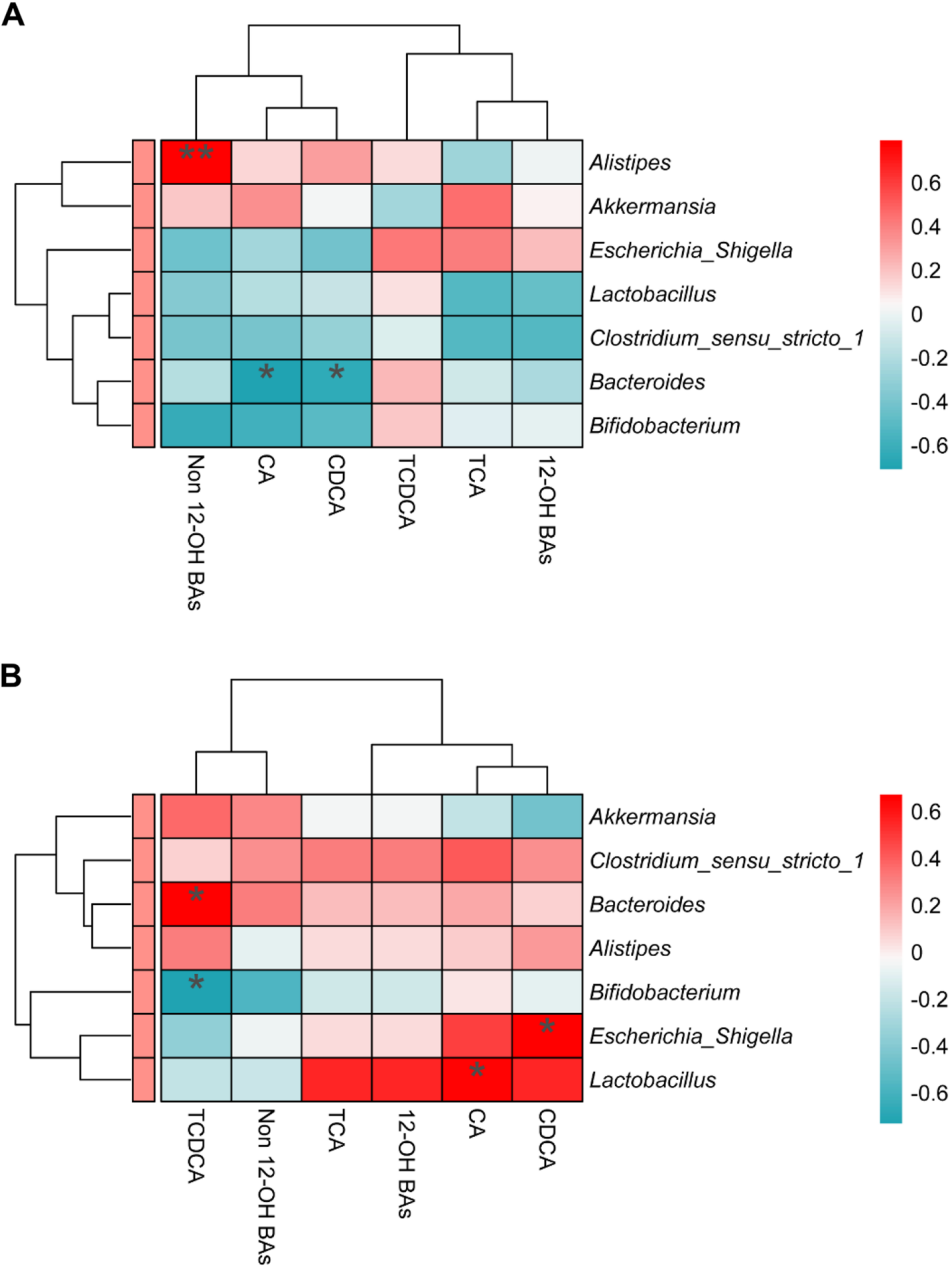
Correlation analysis of gut microbiota and hepatic BAs in broiler chickens supplemented with BAs in LFD (**A**) and HFD (**B**). ^*^P < 0.05, ^**^P < 0.01

To elucidate the relationship between cecum microbiota and BA composition in the liver of broiler chickens, correlation analyses were conducted on the cecum microbiota and BA composition in liver of broiler chickens fed BAs supplemented in LFD and HFD diets. In chickens fed LFD supplemented with BAs, a significant positive correlation was observed between the abundance of *Akkermansia* in the cecum and the content of Non 12-OH BAs in the liver tissue (*P* < 0.05, Fig. 9A). Conversely, a significant negative correlation was found between the abundance of *Bacteroides* in the cecum and the contents of CA and CDCA in the liver tissue (*P* < 0.05, Fig. 9A). In chickens fed HFD with BA supplementation, the abundance of *Bacteroides* in the cecum exhibited a significant positive correlation with the content of TCDCA in the liver tissue, while the abundance of *Bifidobacterium* in the cecum displayed a significant negative correlation with the content of TCDCA in the liver tissue (*P* < 0.05, Fig. 9B). Furthermore, the abundance of *Escherichia* in the gut showed a significant positive correlation with the content of CDCA in the liver tissue (*P* < 0.05, Fig. 9B), and the abundance of *Lactobacillus* in the gut exhibited a significant positive correlation with the content of CA in the liver tissue (*P* < 0.05, Fig. 9B).

## Discussion

The economic viability of broiler chicken production hinges significantly on their fat metabolism status. This study aimed to investigate the impacts of BAs and diets with different fat levels on hepatic fat deposition and growth performance of broiler chicken involvement of gut microbiota and liver BA composition. The findings contribute to optimize the utilization of BAs in feed formulations to enhance growth performance, thereby mitigating fatty liver formation and improve metabolic health in broiler chickens.

It has been established that the composition of broiler chicken feed profoundly affects their growth performance and product quality. Fat serves as a crucial energy source, promoting growth rate and weight gain while enhancing feed utilization efficiency and reducing costs [24]. However, excessive fat intake can disrupt broiler chicken fat metabolism [25]. A study indicated that supplementing feed with BAs can ameliorate hepatic fat deposition induced by high-energy diets and reduce abdominal fat in broiler chickens [26]. Consistent with this finding, our research demonstrates that exogenous BA supplementation decreases hepatic tissue TG content and reduces abdominal fat weight and percentage in broiler chickens fed an HFD, underscoring the role of BAs in regulating broiler chicken fat metabolism. The liver plays a pivotal role in broiler chicken fat metabolism [27]. Addition of BAs to diets can downregulate the expression of hepatic fat synthesis-related genes such as *FAS* and *ACC*, thus mitigating hepatic fat deposition resulting from high-energy feed [26, 28]. Our findings further reveal that BA supplementation in both LFD and HFD reduces the expressions of *FAS* and *ME* in hepatic tissue, indicating a decrease in fat synthesis of liver in broiler chicken. Research indicates that synthesized and excreted BAs in liver tissue play a pivotal role in balancing lipid metabolism and bile system function [29]. Consequently, it raises the question: do exogenous BAs regulate broiler chicken hepatic lipid metabolism by influencing the composition of BAs in hepatic tissue? The liver serves as the principal organ for lipid metabolism of broiler chicken and is the primary site for bile synthesis and metabolism [30]. BAs are predominantly synthesized in the liver through two pathways: the classical or neutral pathway involving CYP7A1 enzyme catalysis and the alternative or acidic pathway involving CYP27A1 enzyme catalysis. These pathways yield different bile acid compositions, with species variations observed [31]. In contrast to other animals, our results indicate that in hepatic tissue of broiler chicken, the primary 12-OH BAs are TCA and CA, while the primary Non 12-OH BAs are TCDCA and CDCA [32]. This is consistent with the reports stating that taurine is the predominant amino acid in chicken bile, comprising 62% of bile nitrogen [33]. Under LFD, TCA accounts for approximately 25% of total BAs in hepatic tissue of broiler chicken, while TCDCA accounts for around 55%, collectively representing over 80% of the total BA content in hepatic tissue, aligning with existing literature on mammalian BAs conjugation patterns [31]. Our findings reveal that supplementing the LFD with BAs significantly increases the Non 12-OH BA/12-OH BA ratio in hepatic tissue, while supplementation of HFD significantly decreases this ratio. CYP8B1, a key enzyme in CA synthesis, plays a crucial role in determining this ratio by influencing the balance between Non 12-OH BAs (e.g., CDCA) and 12-OH BAs (e.g., CA) [34]. Recent studies have shown that alterations in CYP8B1 expression levels can modulate 12-OH BA levels, consequently affecting host metabolism beneficially [35]. Our analysis of key BA synthesis in hepatic tissue indicates that BA supplementation under LFD conditions reduces *CYP8B1* expression, while under HFD conditions, it increases *CYP8B1* expression. This suggests that the regulatory mechanism of BAs on hepatic fat metabolism varies between LFD and HFD conditions.

The result of present study showed that BA supplementation tended to decrease abdominal fat weight (*P* = 0.06) while significantly reduced organ index (*P* < 0.01) at 42 days of age. As no detectable (*P* > 0.05) interaction of diet and BA treatments was observed, the effect of BAs on abdominal fat seems to be independent of diet treatment. Although the absolute muscle weight was heavier in LFD treatment, compared with HFD-chickens, BAs treatment had no significant influence (*P* > 0.05) on breast or thigh muscle weight. The possible explanation is that the energy and protein levels in LFD and HFD were kept the same and the favorable effect of BAs on energy utilization is limited. This speculation is supported by the observation that growth performance was not significantly influenced by BAs. The supplemental effect of BAs on growth performance of broilers fed with high-energy diet remains to be elucidated.

The gut microbiota plays a pivotal role in BA metabolism and recycling, thereby affecting BA concentration and composition [36]. Intestinal microorganisms can produce secondary BAs through various reactions, with the types and structures of these BAs influencing the Non 12-OH BA/12-OH BA ratio [37]. Our experimental results demonstrate that the predominant communities bacterial in the cecum microbiota of broiler chickens include *Alistipes*, *Akkermansia*, *Bacteroides*, *Ruminococcaceae*, and *Faecalibacterium*. Specifically, BA supplementation in LFD increases the abundance of *Akkermansia* while decreasing the abundance of *Alistipes*, whereas supplementation in HFD yields opposite effects. Since the discovery of *Akkermansia*, numerous studies have linked its absence or reduction to various diseases, fatty liver included, underscoring its importance [38, 39]. Conversely, *Alistipes* can utilize available medium- and long-chain fatty acids in the intestinal environment as nutrients, affecting fatty acid proportions [40, 41]. Studies have reported associations between BAs supplementation and alterations in fatty acid concentrations in broiler chicken breast muscles, possibly related to *Alistipes* abundance in the gut microbiota [4, 42]. Moreover, the gut microbiota can participate in BA conversion metabolism, with bacteria encoding bile salt hydrolase (BSH), 7-alpha hydroxysteroid dehydrogenase (7-alpha HSDH), and 7-alpha-dehydroxylase playing key roles [43–45]. Our research results demonstrate that BA supplementation in both LFD and HFD conditions reduces the abundance of *Bacteroides* in broiler chicken while increasing the abundance of *Escherichia*. However, the effects of BAs on *Bifidobacterium*, *Clostridium*, and *Lactobacillus* abundances in broiler chicken guts show contrasting results between LFD and HFD conditions. These findings suggest that certain bacterial may affect BA regulation on hepatic lipid metabolism through the gut-liver axis in broiler chicken. Correlation analyses further indicate significant associations between specific cecum bacterial communities and hepatic tissue BAs content in broiler chickens treated with BAs in LFD. For instance, the abundance of *Akkermansia* correlates positively with Non 12-OH BA content in hepatic tissue, while the abundance of *Bacteroides* correlates negatively with CA and CDCA contents in hepatic tissue. These findings suggest that BAs supplementation in LFD may increase Non 12-OH BA content and the Non 12-OH/12-OH BA ratio of hepatic tissue through *Akkermansia*, thereby reducing hepatic fat deposition in broiler chickens. Non 12-OH BAs may play a pivotal role in hepatic lipid metabolism in broiler chickens fed LFD, and perhaps supplementing diets with Non 12-OH BAs or *Akkermansia* alone can alleviate hepatic fat deposition in broiler chickens, thereby improving growth performance and conserving the use of 12-OH BAs. Conversely, supplemented with BAs in the HFD, correlations between certain gut bacterial strains and hepatic tissue BA content suggest that BA supplementation may affect hepatic tissue TCDCA, CDCA, and CA content through *Bacteroides*, *Bifidobacterium*, *Escherichia*, and *Lactobacillus* in the broiler chicken, consequently reducing hepatic fat deposition. It is well known that dietary fiber has a profound influence on gut microbiota [46, 47]. In this study, the dietary composition of HFD had relative higher level of dietary fiber (LFD: 1–3 weeks, 2.3%, 4–6 weeks, 2.1%; vs. HFD: 1–3 weeks, 3.0%, 4–6 weeks, 2.8%) to keep dietary energy level the same as LFD. Hence, the conclusion should be explained with caution.

For modern line of broiler chickens, the prevailing conditions such as excessive energy intake and low levels of activity made the fatty liver syndrome incidence in broiler breeders [48] and commercial broilers [49, 50]. In the present study, the supplemental effect of BAs on broilers indicated that BAs decreased hepatic lipid content and abdominal fat deposition, in line with previous work [51]. In this study, the experimental diets had different lipid contents and same metabolizable energy level. Further studies are warranted to the supplemental effect of BAs on growth performance and profitability to broiler industry.

## Conclusions

In conclusion, our findings suggest that supplementing BAs could potentially offer beneficial effects on addressing fatty liver issues in broiler chickens. The result suggests that the altered microbiota-bile acid profiles is associated with the changed hepatic lipid metabolism. The result highlights the beneficial effect of BAs on liver health of broiler chickens while maintaining high growth performance under high-fat diet conditions. Future investigations should delve deeper into the application potential of BAs in broilers fed with high-energy diet.

## Abbreviations

ACC: Acetyl-CoA carboxylase
BA: Bile acid
BSH: Bile salt hydrolase
CA: Cholic acid
CDCA: Chenodeoxycholic acid
CYP27A1: Cytochrome P450 family 27 subfamily A member 1
CYP7A1: Cytochrome P450 family 7 subfamily A member 1
CYP7B1: Cytochrome P450 family 7 subfamily B member 1
CYP8B1: Cytochrome P450 family 8 subfamily B member 1
FAS: Fatty acid synthase
HCA: Hyodeoxycholic acid
HFD: High-fat diet
LFD: Low-fat diet
MCA: Muricholic acid
NEFA: Non-esterified fatty acid
SREBP: Sterol regulatory element-binding protein
TBA: Total bile acids
TG: Triglyceride
UDCA: Ursodeoxycholic acid
