# Correction: Dietary bile acids supplementation decreases hepatic fat deposition with the involvement of altered gut microbiota and liver bile acids profile in broiler chickens

**DOI:** 10.1186/s40104-024-01123-3

**Published:** 2024-11-11

**Authors:** Minghui Wang, Kelin Li, Hongchao Jiao, Jingpeng Zhao, Haifang Li, Yunlei Zhou, Aizhi Cao, Jianmin Wang, Xiaojuan Wang, Hai Lin

**Affiliations:** 1 College of Animal Science and Technology, Shandong Provincial Key Laboratory of Animal Biotechnology and Disease Control and Prevention, Key Laboratory of Efficient Utilization of Non-Grain Feed Resources (Co-Construction By Ministry and Province), Ministry of Agriculture and Rural Affairs, Shandong Agricultural University, No. 61, Daizong Street, Taian, 271018 Shandong P. R. China; 2 College of Life Sciences, Shandong Agricultural University, No. 61, Daizong Street, Taian, 271018 Shandong P. R. China; 3 College of Chemistry and Material Science, Shandong Agricultural University, No. 61, Daizong Street, Taian, 271018 Shandong P. R. China; 4 Shandong Longchang Animal Health Products Co., Ltd., Jinan, P. R. China

**Correction:** **J Animal Sci Biotechnol 15, 113 (2024)**

**https://doi.org/10.1186/s40104-024-01071-y**

Following publication of the original article [1], the authors reported a typo in the author name.

The author name **Jianming Wang** should be corrected to **Jianmin Wang**.

The original article [1] has been updated.
